# Factors influencing trends in opioid prescribing for older people: a scoping review

**DOI:** 10.1017/S1463423620000365

**Published:** 2020-09-24

**Authors:** Rasa Mikelyte, Vanessa Abrahamson, Emma Hill, Patricia M. Wilson

**Affiliations:** 1 Research Associate, Centre for Health Services Studies, University of Kent, Canterbury, UK; 2 Sessional GP & Sessional General Practitioner and Honorary Research Fellow, Centre for Health Studies, University of Kent, Canterbury, UK; 3 Professor of Primary and Community Care, Centre for Health Services Studies, University of Kent, Canterbury, UK

**Keywords:** older people, opioids, pain management, prescribing

## Abstract

**Aim:**

The review aimed to identify factors influencing opioid prescribing as regular pain-management medication for older people.

**Background:**

Chronic pain occurs in 45%–85% of older people, but appears to be under-recognised and under-treated. However, strong opiate prescribing is more prevalent in older people, increasing at the fastest rate in this age group.

**Methods:**

This review included all study types, published 1990–2017, which focused on opioid prescribing for pain management among older adults. Arksey and O’Malley’s framework was used to scope the literature. PubMed, EBSCO Host, the UK Drug Database, and Google Scholar were searched. Data extraction, carried out by two researchers, included factors explaining opioid prescribing patterns and prescribing trends.

**Findings:**

A total of 613 papers were identified and 53 were included in the final review consisting of 35 research papers, 10 opinion pieces and 8 grey literature sources. Factors associated with prescribing patterns were categorised according to whether they were patient-related, prescriber-driven, or system-driven. Patient factors included age, gender, race, and cognition; prescriber factors included attitudes towards opioids and judgements about ‘normal’ pain; and policy/system factors related to the changing policy landscape over the last three decades, particularly in the USA.

**Conclusions:**

A large number of context-dependent factors appeared to influence opioid prescribing for chronic pain management in older adults, but the findings were inconsistent. There is a gap in the literature relating to the UK healthcare system; the prescriber and the patient perspective; and within the context of multi-morbidity and treatment burden.

## Introduction

Persistent pain occurs in 45%–85% of adults over the age of 65 (Jakobsson *et al.*, 2004) often with serious impact on function and wellbeing (Gianni *et al.*, 2009). Pain treatment and management are important contributors to the quality of life in older people and facilitate participation in valued activities. Help the Aged (Kumar and Alcock, 2008), a UK charity advocating for older people, reported the views of those living with pain and emphasised the pervasive nature pain has on all areas of life, including other medical conditions, mental health, relationships, opportunities to socialise, and identity all of which are reported elsewhere (Holloway *et al.*, 2000; Reyes-Gibby *et al.*, 2002; Drummond, 2003; Closs *et al.*, 2009).

While common, pain in older people appears to be under-recognised and under-treated (Cavalieri, 2002). Although older people are more likely to experience pain than younger people (Fayaz *et al.*, 2016), older people are less likely to receive effective and sufficient help for their pain (Makris *et al.*, 2015) partly due to inadequate assessment. Pain assessments are disproportionately overlooked for older people, with some physicians perceiving pain as a normal part of ageing (Niemi-Murola *et al.*, 2007). Additionally, pain in older people often presents ‘atypically’, for example, poorly localised and lasts longer compared to younger counterparts (Robinson, 2007). Given that pain assessments as well as physician training in pain management are based on studies of younger (Peters *et al.*, 2007), often male (Samulowitz *et al.*, 2018), identifying and recognising pain in older people can be challenging. Furthermore, older people are less likely to vocalise their pain, experience greater self-doubt around reporting it, and may use different words (e.g., ‘sore’ or ‘aching’) to describe pain compared to younger people (Collett *et al.*, 2007).

The issues around assessing pain are exacerbated in residential and nursing homes where access to physicians is often less frequent than in a community setting (Ferrell, 2004; Hunnicutt *et al.*, 2017). Additionally, residents often have cognitive and/or communication difficulties (Frampton, 2003), which is problematic given that the majority of widely used and standardised pain assessments rely on self-reporting.

Pain management also presents age-related issues. The ageing process *per se* increases sensitivity to both the intended and unintended effects of pain medication (Beyth and Shorr, 2002). Multi-morbidity and polypharmacy, both common among older people (Wehling, 2009), can introduce complex drug interactions that exacerbate other health conditions (Marengoni and Onder, 2015). For example, drugs providing pain relief can negatively affect other health conditions such as gastritis. Furthermore, changes in metabolism in later life and the long-term effects of using pain medication over decades (Alam *et al.*, 2012) need to be factored into prescribing decisions (Gloth, 2001). Therefore, as well as under-treatment of pain, over-prescription of strong pain killers, based on the doses required for younger people, larger than those required for older people, adds to the complexity of inappropriate pain management for older people.

Inappropriate prescription of opioids for older people appears particularly prevalent (West and Dart, 2016; Fain *et al.*, 2017). Initiation of strong opioids without first treating pain with simple analgesics or weak opioids has been identified in one-third of community-dwelling older outpatients (Gadzhanova *et al.*, 2013). Prescribing strong opioids is not only more prevalent for older people, but also increasing at the fastest rate in this age group (Roxburgh *et al.*, 2011). Häuser *et al.* (2014) compared the consumption of prescribed opioids for non-cancer pain in 2014 in Australia, Canada, Germany, and the USA and found ‘signs of an opioid epidemic’ (page e-599/p1) in North American and Australian but not Germany and attributed this not to opioids *per se*, but how they are used, ‘without appropriate indication, appropriate precautions, and with excessive doses, often as a monotherapy’ (page e-599/p10).

This trend is problematic given the international ‘opioid crisis’, or increasing rates of opioid addiction and opioid-related mortality (Dhalla *et al.*, 2011). Opioid-based pain management has a specific impact on older people who may be experiencing falls, memory problems, and incontinence all of which can be exacerbated by opioids (Gianni *et al.*, 2009; Gordon *et al.*, 2010; Morley, 2017). The majority of findings on changes in opioid prescribing for older people come from outside the UK and little is known about the UK-specific context or if the trends in other countries are mirrored in the UK.

Most health and care services in the UK are commissioned by groups of GP Practices known as Clinical Commissioning Groups (CCGs) and our local CCG (Canterbury and Coastal) provided the catalyst for this study. There is little UK literature concerning not only *what* the trends of inappropriate opioid prescribing for older people are, but also *why* inappropriate prescribing occurs in this age group (and compared to younger people). This scoping review aimed to ascertain what factors influence opioid prescribing as non-palliative pain-management medication for older people; the results will be used to inform practice development and training within the CCG.

## Methods

A scoping literature review is a comprehensive and systematic approach that allows for a broad research question and incorporates all sources, including grey literature, compared to a standard systematic review that focuses on a ‘narrow range of quality assessed studies’. We used Arksey and O’Malley’s (2005) framework as it offers a rigorous approach suited to identifying gaps in existing literature (Reyes-Gibby *et al.*, 2002) and reviewing areas that are complex and broad. It comprises five stages: identifying the research question (as above); identifying relevant studies; study selection; charting the data; and collating, summarising, and reporting the results.

### Identifying relevant studies

The review was guided by the following inclusion and exclusion criteria:

#### Inclusion criteria

1. Literature, including all study designs and publication types, from Peer-Reviewed Journals from January 1990 to September 2017
2. Grey literature (e.g., policy papers) from January 1990 to September 2017
3. Literature in English language only (resource restrictions meant that translation services could not be used)
4. Papers that involve older adult participants (i.e., participants aged 65 and older) regardless of setting (i.e., community-dwelling older people as well as those living in care/nursing homes), or looked at external perceptions of older adult pain management
5. Papers on opioid prescribing for pain management for older people

#### Exclusion criteria

1. Bachelor and Masters dissertations; unpublished doctoral theses
2. Papers specifically focusing on opioid use, rather than prescribing (studies focusing on use/misuse of opioids were outside of the scope for this review, which concerned factors influencing *prescribing* of opioids)
3. Papers specifically assessing methodological instruments or approaches (e.g., efficacy of risk minimisation tools in opioid prescribing)
4. Papers on palliative or end-of-life care
5. Papers on opioids as substitution (e.g., for heroin) rather than pain management
6. Guidelines addressing how clinicians *should* prescribe, rather than what affects current prescribing

**Figure 1.**
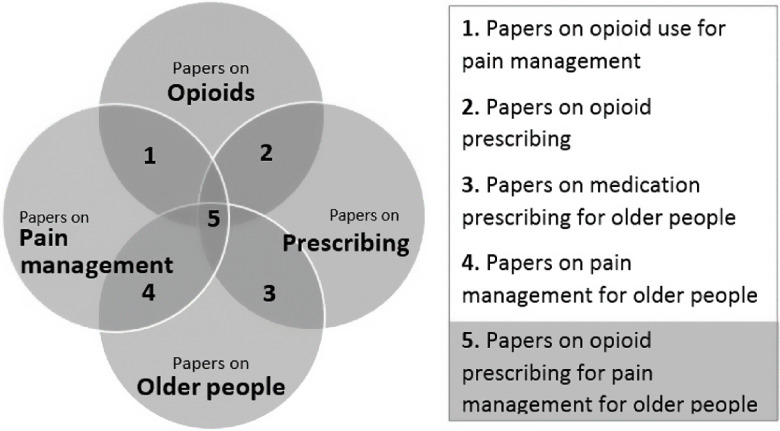
The conceptual framework guiding the literature search

A conceptual diagram was developed to focus the literature search on the intersection of 4 topics (see Area 5 of Figure 1). The search terms were developed for electronic databases (PubMed, EBSCO Host, and Google Scholar). The UK Drug Database, a general practitioner and a palliative care clinician were consulted to ensure no specific types of opioids were excluded from the search. The search terms/keywords were:

- ‘opioid’ *or* ‘opiate’ *or* ‘oxycodone’ *or* ‘oxycontin’ *or* ‘fentanyl’ *or* ‘hydrocodone’ *or* ‘Co-dydramol’ *or* ‘hydromorphone’ *or* ‘meperidine’ *or* ‘pethidine’ *or* ‘morphine’ *or* ‘codeine’ *or* ‘alfentanil’ *or* ‘dihydrocodeine’ *or* ‘diamorphine’ *or* ‘meptazinol’ *or* ‘pentazocine’ *or* ‘papaveretum’ *or* ‘remifentanil’ *or* ‘buprenorphine’ *or* ‘tramadol’ *or* ‘tapentadol’ *or* ‘dipipanone’ *or* ‘buprenorphine’ **AND**
- ‘older adult’ *or* ‘older person’ *or* ‘older people’ *or* ‘elders’ *or* ‘elderly’ *or* ‘geriatric’ **AND**
- ‘prescription’ *or* ‘prescribing’ *or* ‘prescribed’ **AND**
- ‘pain management’ *or* ‘pain’

Existing systematic reviews were used to identify primary research. Grey literature was identified both within the above searches and by searching the archives of relevant ‘grey’ journals such as Adverse Reaction Research periodical.

Academic and grey literature resulting from the literature search was then screened: first by title and abstract, and then by reading the full text to determine if inclusion/exclusion criteria were met. A data extraction tool was used for full-text review. Reference lists of reviewed articles were also scanned of relevant papers.

Two authors (R.M. and V.A.) carried out study selection. At each stage of selection, the authors first worked together to establish a consistent approach, and then independently. Sources were categorised into those that should be included in the final synthesis, those that did not meet the inclusion criteria, and those where both researchers were uncertain. A third researcher (P.W.) independently reviewed papers where the primary reviewers remained uncertain.

Papers meeting selection criteria were then summarised in Table 1, extracting specifically study setting, methodology, sample characteristics, study aims/objectives, and findings informing which factors influenced opioid prescribing for older people. These sources were further categorised by (1) whether the source suggested that opioids were being underprescribed, overprescribed, demonstrated complex prescription patterns, or had no explicit stance; (2) factors associated with different prescribing trends (e.g., higher versus lower opioid prescribing); and (3) whether influencing factors were patient characteristics, prescriber characteristics, or policy/system factors. A narrative framework (Arksey and O’Malley, 2005) was used to synthesise the findings, analyse knowledge gaps, and identify areas of consensus or disagreement.

**Table 1.** Studies reporting factors influencing opioid prescribing for pain-management in older people

Author	Setting	Methodology	Total Number in Sample	Aim/Objectives	Relevant Findings on Influencing Factors
*Research Studies*					
Axmon *et al.* (2017)	Sweden; three national prescribing registers	Retrospective Database Analysis	7936 people with intellectual disabilities and 7936 controls (aged 55–96)	To describe the prescription of potentially inappropriate medications among older people with intellectual disabilities in relation to prescriptions among their age peers in the general population	People in the Intellectual Disability (ID) cohort were less likely to be prescribed tramadol than those in the general population (controls), but there was no difference between the ID and general population cohorts with regards to the number of years with a prescription
Bell *et al.* (2011)	Finland; community-dwelling individuals	Retrospective Database Analysis	28 093 people with Alzheimer’s disease and 28 093 matched controls (aged 42–101)	To investigate the national pattern of strong opioid use among community-dwelling persons with and without Alzheimer’s disease (AD) in Finland	Although the overall use of opioid analgesics was lower among persons with Alzheimer’s disease compared with those without, the use of strong opioids was higher. This was due to the higher use of transdermal fentanyl among persons with Alzheimer’s disease
Boerlage *et al.* (2008)	Rotterdam, Netherlands; residential homes only	Questionnaire	183 care home residents (without cognitive impairment; age range not reported; median age 88)	To investigate the prevalence of pain in elderly people living in Dutch residential homes, pain characteristics and intensities, type of analgesics prescribed, and impact of pain on the residents’ daily functioning	Of the residents with pain, 22% did not receive any analgesics and only 3% were prescribed a strong opioid. When analgesics were prescribed, 31% of residents received them only ‘as needed’. Almost 60% of residents agreed that pain was part of ageing and 72% indicated that they did not always report pain to caregivers. Satisfaction with caregivers’ and doctors’ attention to pain was 37% and 39%, respectively
Bradley *et al.* (2012)	Northern Ireland	Retrospective cross-sectional population study	166 108 persons (aged ≥70 years)	To investigate potentially inappropriate prescribing in an older population in a neighbouring jurisdiction (Northern Ireland), using primary care data	Polypharmacy in older adults increases the risk of potentially inappropriate prescribing, including that of opioids
Closs *et al.* (2004)	North of England	Questionnaire (user perspective)	113 nursing home residents from 15 nursing homes (aged 56–103)	To explore analgesic prescription and administration according to nursing home residents’ cognitive status	Those with greater cognitive impairment received fewer analgesics (including opioids) than others
Fain *et al.* (2017)^25^	US care homes	Retrospective Database Analysis	18 526 nursing home residents (without cancer, cognitive impairment or palliative illness; aged ≥65)	To quantify prescription analgesic use of elderly nursing home residents with persistent non-cancer pain and to identify individual and facility traits associated with no treatment	Controlling for other variables, older age, greater cognitive impairment, and self-pay status was statistically significantly associated with no analgesic prescribing (including opioids) among nursing home residents
Gadzhanova *et al.* (2013)	Australia, a veteran community	Retrospective Database Analysis	10 791 people who were initiated with oxycodone in 2010 (veterans of any age)	To explore patterns of non-opioid, weak opioid, and strong opioid use prior to initiation of oxycodone for non-cancer pain in a predominantly older Australian population	Oxycodone is frequently initiated for non-cancer pain without first trialling other analgesics. There is a lack of attempt to use other, weaker analgesics first, before initiating strong opioids
Gianni *et al.* (2010)	Italy, eight geriatric hospital departments	Questionnaire (GP and user perspective)	367 patients (age range in the geriatric unit not reported, but the average age was 78)	To evaluate the incidence of pain in patients recovering in acute geriatric units, and the perceived effectiveness of pain management strategies in relation to demographic and psychosocial characteristics	Only 49% of those with pain had any type of treatment, which was adequate for the pain intensity and 74.5% of patients considered the therapy to be of low or no efficacy. Strong opioids were used only in 4.4% of patients with a pain score of 9 and in 36.4% of patients with a pain score of 10, but were not used at all for pain score < 9. The level of pain *did not* explain prescribing
Goulding (2004)	USA	Retrospective Survey Analysis	13 003 older patient visits to physician offices (aged ≥65)	To examine the relationship of patient, visit, and physician characteristics with inappropriate prescribing. To explore the higher risk of inappropriate prescribing that has already been associated with female patients	Gender is associated with opioid prescribing for older people. For visits in which a pain reliever was prescribed, the proportion with an inappropriate pain reliever was higher for elderly females than for elderly male visits (10.8% versus 5.9%; *P* <0.001)
Green *et al.* (2013)	North Staffordshire, UK	Questionnaire	873 (19%) of community-dwelling older people (aged ≥50) with joint pain prescribed opioids (out of 4652 in the dataset)	To explore the association of patients’ socio-demographic and lifestyle factors, and pain severity, physical functioning, mental health and social participation with prescription of high-strength painkiller medication over 3 years	For over 50s with joint pain increased opioid prescription over the 3 years associated with being younger, male, severe joint pain, poor physical function, and lower frequency of alcohol consumption
Griffioen *et al.* (2017)	Netherlands	Questionnaire	435 physicians (324 ECPs and 111 in ECP trainees; aged 25–66)	To evaluate the knowledge and attitudes of elderly-care physicians (ECPs) and ECP trainees towards prescribing opioids in long-term care facilities	Physicians working in long-term care in the Netherlands have a positive attitude towards opioids and do not consider addiction a reason to refrain from the clinical use of opioids. The most important barriers to opioid prescriptions were reluctance of patients to use opioids (83%), unknown degree of pain (79%), and pain of unknown origin (51%)
Iyer (2011)	USA	Cross-sectional analysis	5661 Emergency Department visits (2003–2006) by patients aged ≥65	To determine the patient characteristics associated with differences in pain score documentation and to evaluate the association between these characteristics and analgesic use in the Emergency Departments	Failure to assess pain limits the ability to treat pain. As patient age increases, the likelihood of having a pain score documented drops significantly. Documentation of a pain score was associated with increased odds of an analgesic, and opioid, prescription when a pain score was designated. Although gender and race were not predictors of pain documentation, they were predictors of analgesic prescription; African Americans were underprescribed opioid analgesics (but not non-opioid analgesics) and women were prescribed less of any analgesics
Jensen-Dahm *et al.* (2015)	Denmark, community and nursing homes	Cross-sectional database analysis	35 455 older people with dementia and 870 645 nursing home residents without dementia (aged ≥65)	To explore if frail elderly such as patients with dementia and/or nursing home residents were less likely to receive opioids and particularly strong opioids	Nursing home residents used opioids most frequently (41%), followed by home-living patients with dementia (27.5%) and home-living patients without dementia (16.9%)
Kaasalainen *et al.* (2007)	Canada	Semi-structured interviews and focus groups	9 GPs and 57 nurses in 4 care/nursing homes (age range not reported)	To explore attitudes and beliefs that affect decisions about prescribing and administering pain medications in older adults who live in long-term care, with a particular emphasis on those with cognitive impairment	Physicians and nurses were reluctant to use opioids, and some physicians acknowledged that they would not prescribe opioids to residents under any condition. The reasons for underutilisation of opioids in older adults were the poor quality of pain assessments and concerns over polypharmacy, opiophobia, addiction, and other adverse effects
Karp *et al.* (2013)	Pittsburgh, USA	Descriptive, hypothesis-generating, longitudinal study	1109 older people; biennial assessment waves (aged ≥65)	To examine a variety of clinical and demographic baseline characteristics and their associations with longitudinal patterns of opioid and non-opioid analgesic use in a population-based cohort of rural older adults	Predictors of long-term use of both opioid and non-opioid analgesics included female, taking 2 or more prescription medications, and ‘arthritis’ diagnoses. Long-term opioid use was also associated with age 75–84 years
Maiti *et al.* (2018)	Tertiary care facility, New York, USA	Retrospective cohort study of patient medical records	9245 patients (aged ≥65)	To describe patterns of opiate prescribing and associated outcomes in hospitalised older adults	There was no difference in sex, race, ethnicity, or Charlson Comorbidity Index between opiate exposure groups (no opiates versus prior opiates versus new opiates)
Manias (2012)	Australia, two geriatric units	Observational study	34 nurses (aged 22–60) 285 patients (aged 62–96)	To examine how pain was assessed and managed in older patients who were admitted to geriatric units	Clinical practice complexities, such as de-prescribing policy, quick and simple verbal assessments of pain, and subjective judgements of ‘tolerable’ pain were important in explaining prescribing patterns
Marengoni and Onder (2015)	Italy	Retrospective Database Analysis	1332 patients (aged ≥65) enrolled in 2008; 1380 in 2010; and 1340 in 2012	To evaluate the prevalence and type of analgesic and opioid prescriptions at hospital admission and discharge in elderly patients, factors associated with an opioid prescription, and the relationship between presence of pain and analgesic prescription at hospital discharge	The number of total prescribed drugs was positively associated with opioid prescriptions in the three runs; in the third, dementia and a better functional status were inversely associated with an opioid prescription. The conservative attitude of Italian physicians to prescribe opioids in elderly patients changed very little between hospital admission and discharge through a period of 5 years
Morrison and Siu (2000)	New York, USA	Questionnaire Study and Medical Record Review	59 cognitively intact elderly patients with hip fracture and 38 patients with hip fracture and advanced dementia (all aged ≥70)	To examine the treatment of pain following hip fracture in cognitively intact patients and those with advanced dementia	The majority of elderly hip fracture patients experienced undertreated pain. 83% of cognitively intact patients and 76% of dementia patients did not receive a standing order for an analgesic agent. The advanced dementia patients received one-third the amount of morphine sulphate equivalents as of the cognitively intact patients
Niemi-Murola *et al.* (2007)	Helsinki, Finland	Questionnaire Study	430 medical students (out of 680/response rate 63%; age range not reported, but the average age was 24)	To construct an instrument/questionnaire and to assess the effect of pain education on medical students’ attitudes at the University of Helsinki	Final year students felt significantly more often anxious about seeing a chronic pain patient (*P* < 0. 05) compared with the first-year students. Female students were more anxious about seeing a patient suffering from chronic pain (*P* < 0.05) and they were less confident of their ability to treat chronic pain patients in primary care (*P* < 0.001) than the male students. The age of the medical students (i.e., being older) correlated significantly with not seeing pain as a normal part of ageing and willingness to prescribe opioids for cancer pain
Petre *et al.* (2012)	Baltimore, USA	Retrospective Database Analysis	352 patients selected for elective hip or knee replacement (2005–2008; two age groups: 60–79 and ≥80)	To explore whether there are age-related differences in pain, opiate use, and opiate side-effects after total hip or knee arthroplasty in patients 60 years or older	Older patients (80 years or more) had significantly fewer opiates prescribed but significantly more side-effects, including delirium, than the younger patients (60–79 years), even after adjusting for opiate dose and pain score
Pittrow *et al.* (2003)	Berlin, Germany	Retrospective Database Analysis; Longitudinal Study	996 nursing home residents (aged ≥60)	To describe the prescription pattern of physicians for nursing home residents and to compare the prescriptions issued by nursing home-based physicians with those by office-based physicians	There was no difference in opiate prescribing in both groups (nursing home-based physicians versus office-based physicians)
Ponteand & Johnson-Tribino (2005)	West Virginia, USA	Survey of family physicians	185 useable surveys out of 537/response rate 34.5% (age range not reported)	To determine physicians’ attitudes, beliefs, and knowledge regarding evaluation and treatment of pain	The majority of respondents (80.0%) were not apprehensive about prescribing high-dose opioids to patients with chronic malignant pain but were anxious about prescribing them to those with chronic non-malignant pain. Most did not administer opioids to individuals with a history of substance abuse. Nearly two-thirds indicated that scrutiny by regulatory agencies affected their prescription of opioids. Approximately 60% felt that their formal medical training did not prepare them to effectively manage pain, and exhibited substantial knowledge gaps
Raji *et al.* (2017)	Texas, USA	Retrospective Database Analysis	Sample size not specified; medication claims 2007–2012 for people aged ≥66	To examine the longitudinal association of laws regulating pain clinics on opioid-prescribing and opioid-related toxicity among Texas Medicare recipients	There was a short-lived decline in the monthly percentages of patients who filed a schedule II or schedule III opioid prescription after the 2009 laws regulating pain clinics. The decline lasted about three months. Subsequent new laws had no effect on the percentages of patients who filed any opioid prescription or were hospitalized for potential opioid toxicity
Rognstad *et al.* (2013)	Norway	Cluster Randomised Controlled Trial of continuing medical education (CME) groups	Intervention data for 41 CMEs (250 GPs), control data for 39 CMEs (199 GPs), age range not reported	To study the effects of a multifaceted educational intervention on GPs’ Potentially Inappropriate Prescriptions for older patients	Twenty-nine percent decrease in Potentially Inappropriate Prescriptions of opioids and spasmolytics for intervention group where GPs received additional education
Ruiz *et al* (2010)	Florida, USA	Three focus groups and nine semi-structured interviews	14 GPs (aged 34–66) and 5 nurses (aged 55–69)	To explore and understand prescriber attitudes towards, and experiences with, managing chronic non-malignant pain in veterans and using a pain clinical decision support system	In relation to opioid prescribing, clinicians were concerned about side-effects specific to ageing such as impaired motor function and cognition, institutional restrictions on the type and amount of opioids that could be prescribed. Addiction was an infrequent concern for older patients compared to younger counterparts
Sandvik *et al.* (2016)	Norway, nursing homes	Secondary data analysis of three cross-sectional studies from 2000, 2004, and 2011, and an RCT in 2009	2000 (*n* = 1926), 2004 (*n* = 1163), and 2011 (*n* = 1858) and an RCT in 2009 (*n* = 850) (age range not reported, but mean age across all cohorts was 84–85.5 years)	To investigate the prescribing patterns of scheduled analgesic drugs in Norwegian nursing home patients from 2000 to 2011, and association with age, gender and cognitive function	Strong opioid prescriptions increased from 1.9% in 2000 to 17.9% in 2011, whereas non-steroidal anti-inflammatory drug prescriptions decreased from 6.8% to 3.2% in the same period. In 2000, 2004, and 2009 (but not 2011) people with dementia received fewer analgesics compared with patients without dementia
Shah *et al.* (2012)	UK, nursing homes	Retrospective Database Analysis	10 387 residents in care homes and 403 259 older people in the community (aged 65–104)	To compare prescribing quality in care homes in England and Wales with the community and with US, nursing homes	Thirty-three per cent of care home residents in England and Wales received potentially inappropriate medication, including opioids, compared to 21.4% in the community. The potentially inappropriate prescribing rate in US, nursing homes was similar to England and Wales
Shega *et al.* (2006)	Chicago, USA	Cross-sectional analysis of an observational cohort study	115 patient–carer dyads of mostly African American community-dwelling people with dementia and their carers (age range not reported, but average age 84.1)	To explore the pharmacological treatment of non-cancer pain in persons with dementia and identify predictors associated with insufficient analgesia	No patients had been prescribed a Class III (strong opioid) drug. Insufficient analgesia was associated with greater age, Mini-Mental State Examination score of <10, and impairment in daily functioning. Insufficient analgesia was 1.07 times as likely for each additional year of age, 3.0 times as likely if the subject had advanced dementia, and 2.5 times as likely if the patient had any impairment in activities of daily living
Shugarman *et al.* (2010)	Virginia, USA	Clinical vignette review of elderly frail patient and follow-on survey	Of 208 (74%) responding practitioners, 189 with prescribing responsibilities (age range not reported, but average age 42.5)	To evaluate factors associated with practitioners’ intention to address diverse aspects of pain	Factors associated with greater intent to prescribe an opioid included female gender, being an attending physician, being a primary care clinician, and greater confidence in pain management skills. Prescribing opioids was less likely if perceived as an administrative burden
Solomon *et al.* (2006)	Pennsylvania, USA	Retrospective Database Analysis of healthcare beneficiaries (1996–2001)	18 099 elderly patients likely to have chronic pain due to osteoarthritis, rheumatoid arthritis or low back pain (aged ≥65)	To examine patterns of chronic opioid use (defined as at least six 30-day prescriptions in a year) in patients with a comparison group of those with ischaemia heart disease	Participants with rheumatoid arthritis were always more likely to be using opioids than the other patient groups, although long-term opioid use in older adults was uncommon overall. Low-potency short-acting opioids were the most commonly prescribed analgesics for chronic users from all patient groups. The prior use of medicines for psychiatric illness was associated with long-term opioid use across all diagnosis groups but patients with a prior psychiatric diagnosis were *less* likely to receive long-term opioids. A greater number of physician visits were associated with long-term opioid use. African American ethnicity was associated with a lower probability of receiving long-term opioids. Residence in a nursing home was associated with long-term opioid use in several of the subject groups
Spitz *et al.* (2011)	New York, USA	Focus Group Study	23 physicians and 3 nurse practitioners (aged 28–60)	To describe primary care providers’ experiences and attitudes, as well as perceived barriers and facilitators, to prescribing opioids to treat chronic pain in older adults	Providers perceived multiple barriers to prescribing opioids to older adults with chronic pain and used these medications cautiously. Key barriers were fear of causing harm (77%) and the subjectivity of pain (62%). Others included lack of education; problems converting between opioids; concerns about opioid abuse, addiction or dependence; and patient/family reluctance to try an opioid. Facilitators included studies confirming treatment benefit; patient/family education; validated tools for assessing risk and/or dosing for comorbidities; and palliative care settings (versus chronic pain)
Veal *et al.* (2014)	Australia, care facilities	Retrospective Database Analysis	7309 medicine reviews (age range not reported, but average age 84.4)	To explore the use of analgesics among elderly care home residents, including independent predictors of analgesic use, evaluating analgesic use against pain management guidelines, and identifying potential medication management issues	Many residents (28.1%) were regularly prescribed opioids. They were more likely to be prescribed to females, and those with a history of musculoskeletal pain or pain not otherwise specified, history of fractures, osteoporosis or taking anxiolytics/hypnotics, or other sedating agents. Patients taking opioids were more likely to have a history of cancer; regular dose opioids and optimised paracetamol were more likely to be taken by patients with congestive heart failure. Patients with dementia were less likely to receive optimised paracetamol, opioids, or regular dose opioids
West and Dart (2016)	USA, poison centres	Retrospective Database Analysis	57 681 misuse cases reported to participating in US, poison centres (two age groups: 20–59 and ≥60)	To examine recent trends in the misuse of prescription opioids and associated medical outcomes among older-aged adults (60+ years) and compare the patterns with trends among younger-aged adults (20–59 years)	Population rates of misuse of prescription opioids were higher for older adults than for younger adults, and this disparity increased over time
Won *et al.* (2004)	USA (13 states)	Retrospective Database Analysis	21 380 nursing home residents (aged ≥ 65) with persistent pain	To determine the prevalence of analgesic use, their prescribing patterns, and associations with particular diagnoses and medications in patients with persistent pain	Persistent pain was identified in 49% of residents. One-quarter received no analgesics. There was a lower use of opioids by men than women and by African Americans than whites
*Academic Contributions (Non-Research)*					
Gold (2017)	USA	N/A	N/A	Opinion piece on opioid prescribing for older people amidst a ‘national opioid epidemic’	The author’s starting point is to consider the patient’s goals and how pain impacts on daily life. She argues that if mobility declines due to pain (and without analgesia), the impact on health might be greater than prescribing an opioid at an appropriate dose and with close supervision, given that non-steroidal anti-inflammatories (NSAIDs) have greater side-effects than opioids on blood pressure and kidney function. (Greater) confusion and delirium in dementia patients may be a result of untreated pain
Kress *et al.* (2014)	Europe and USA	N/A	N/A	To outline the extent of untreated pain in this population and the consequent reduction in quality of life, before articulating the reasons why it is poorly or inaccurately diagnosed	Pain is frequently under-reported and under-treated with a variety of consequences for patients. Medical professionals need more education (e.g., about polypharmacy/drug interactions, routes of administration, assessment) and to communicate better with patients and carers to improve adherence. Suggests using a standardised observational tool to assess pain in dementia
Morone and Weiner (2013)	USA	N/A	N/A	To comment on the need for more pain education for clinicians	Clinicians have insufficient training and a one-dimensional (sensory) view of pain which influences their prescribing choices
Podichetty *et al.* (2003)	USA	N/A	N/A	To review the management of musculoskeletal pain syndromes in older adults emphasising the potential role of opioid agents in carefully selected patients	Comorbidities may limit therapeutic choices, particularly in the elderly, where opioids may not be suitable
Prostran *et al.* (2016)	USA	N/A	N/A	To discuss the use of opioids for pain management in older adults	The discrepancy between existing guidelines and lack of knowledge on opioid safety specifically for older adult populations may affect prescribing decisions (the author does not specify in what way but suggests this may create variability of practices)
Robson-Lane and Booker (2017)	USA	N/A	N/A	To provide a framework for the culturally responsive treatment of pain in Black older adults	Black older adults are not as likely as White, non-Latino older adults to discuss pain concerns and are also less likely than White individuals to have a prescription for long-acting opioid drugs. Cultural sensitivity is required for pain management
Schofield (2014)	UK	N/A	N/A	To comment on current evidence and future directions on assessment and management of pain in older adults	Limited evidence on non-pharmacological pain treatments, specifically for older adults, limits treatment options. Under-treatment of pain in care homes may result from reluctance to report pain to care home staff; acceptance by residents and staff that pain is normal for older patients; the low expectation of success from medical staff and concern about side-effects; fear of chemical/pharmacological dependence, and/or addiction; lack of awareness of potential strategies to manage pain
Shega *et al.* (2007)	USA	N/A	N/A	To discuss pain management in cognitively impaired older adults	Cognitively impaired adults may be at greatest risk of poor pain control due to under-recognition and under-treatment of pain. Although people with dementia may perceive pain normally, they may not be able to communicate it effectively. Behaviours associated with dementia, such as challenging behaviour may be a manifestation of pain. Analgesics, including opioids, may cause symptoms that overlap with those of dementia including depression and delirium, thus the risks and benefits must be carefully evaluated
Smith and Bruckenthal (2010)	USA	N/A	N/A	To synthesise and discuss implications of opioid analgesia for medically complicated patients	Age interacts with medically complicated conditions (e.g., renal failure) and makes opioid treatments riskier in terms of their impact on the medical complexities beyond pain
Van Ojik *et al.* (2012)	Netherlands	Literature search	N/A	To determine the appropriateness of the strong-acting opioids buprenorphine, fentanyl, hydromorphone, methadone, morphine, oxycodone and tapentadol in elderly patients	No differentiation can be made between the appropriateness of buprenorphine, fentanyl, hydromorphone, morphine, and oxycodone for elderly patients. Methadone is the only opioid contraindicated in patients with long QT interval syndrome and should be given with caution to those at risk of developing prolongation of the QT interval. Because of the long elimination half-life of methadone, there is a risk of drug accumulation in the long-term treatment of chronic pain
*Grey Literature*					
Best Practice Advocacy Centre New Zealand (2008)	New Zealand	N/A	N/A	To outline several key principles that should be considered when prescribing pain relief for older people	A good education for prescribers is essential for good pain relief in osteo-arthritis, especially in the case of opioids. There is a reluctance to treat non-malignant pain with opioids. Prescribers often incorrectly believe that NSAIDs are safer than opioids
Canadian Institute for Health Information (2017)	Canada	Retrospective Database Analysis	Not specified	To synthesize and explain Pan-Canadian trends in the prescribing of opioids, 2012–2016	Older people are prescribed more opioids than their younger counterparts. Prescribing is inversely linked to risk of harm: older people who are at most risks for opioid-related harm were prescribed strong opioids long-term most often – about one in eight of those prescribed an opioid were prescribed a strong one on a long-term basis
Cook (2016)	USA	N/A	N/A	To discuss the effects of policy change on opioid use	Changing policy and more restrictions on opioid prescribing will mean that older people with chronic pain will be forced to change the way they treat the aches and pains of growing old
Drugs & Therapy Perspectives (2006)	USA	N/A	N/A	To discuss ways to address opioid under-usage for chronic pain in elderly patients	Under-prescribing of opioids in older adults may be a result of patient reluctance to disclose pain; poor communication (dementia, hearing impairment, dysphasia, cultural, and educational differences); prescriber concern with polypharmacy; prescriber opioid-phobia; tolerance; dependence, and addiction; lack of guidelines on opioid use for pain that is neither neuropathic nor nociceptive
Express Scripts Lab (2014)	USA	N/A	N/A	To explore opioid use among older adults (65+) in the USA and to identify ways to improve safe prescribing	There was a 4.5% increase in the use of only opioids for pain management in older adults, between 2009 and 2013. During that same timeframe, the number using only NSAIDs declined by 5.1%. Older people had the highest prevalence of opioid use (8.9% of those aged 65+ in 2013) Thirty percent more women than men took prescription opiates in 2013, which might reflect a higher prevalence of painful chronic conditions such as fibromyalgia. However, men consumed higher amounts of these medications. In the elderly, more women than men take extremely high doses of opiates
Lynch (2011)	USA	N/A	N/A	To provide guidelines on the management of drug–drug interaction in older adults receiving opioid treatment	Multiple medical conditions, polypharmacy, communication problems, poor adherence, inappropriate prescription, and poor continuity of care may impact opioid therapy
Peschin and Bens (2016)	USA	N/A	N/A	To comment on the 2016 draft Centers for Disease Control and Prevention Guideline for Prescribing Opioids for Chronic Pain	Authors comment on limitations in the evidence base with regards to identifying chronic pain patients for whom long-term opioid treatment will be most effective; those most at risk of developing a physical dependence on opioids; and those who will experience reduced tolerance while on long-term opioid treatment
Siciliano (2006)	USA	N/A	N/A	To discuss issues around pain management (acute and chronic) in elderly adults. Issues included assessment; differential diagnosis of pain and dementia; and treatment options	There are multiple barriers to effective pain management in adults with dementia which were divided into three major categories: (a) Patient barriers: patients’ knowledge and attitudes around ageing, illness, opioids, and pain act as barriers, especially with older people who may regard pain as a normal part of ageing. Cognitive impairment may reduce the ability to express pain and fear of side-effects may result in a reluctance to accept opioids for pain relief (b) Professional barriers: knowledge and attitudes towards pain management may reduce appropriate prescriptions. Knowledge relates to the physiology of pain; the clinical pharmacology of opioids; newer treatments and combinations with other procedures; and fears around side-effects, dependence, and addiction. Attitudes relate to the priority attached to pain and the accuracy of patient reporting; the perception that pain management is difficult so best avoided; and that pain must be sufficiently severe to warrant medication (c) Systemic barriers: acute health care is not held responsible for long-term pain management; care pathways lack integration and multiple providers do not co-ordinate their approach including that of pain management which may even be overlooked. In rural areas, access to expertise may be limited

## Findings

### Study selection

**Figure 2.**
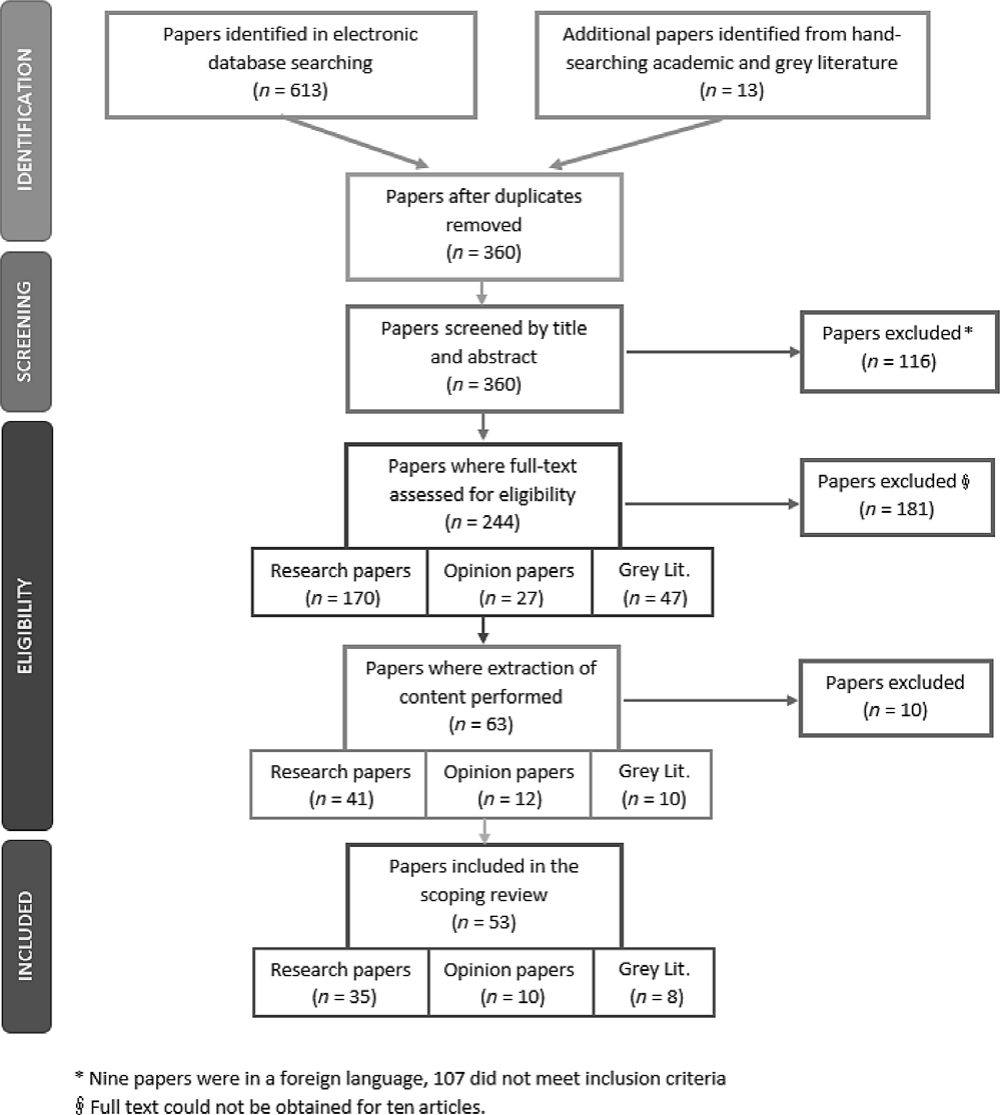
Screening flowchart

The initial search identified 626 papers with 360 remaining once duplicates were removed. These papers were screened by title and abstract; 116 were excluded, leaving 244 to assess by reading full text. Nine of the 116 excluded sources were non-English language. Figure 2 summarises the process.

Of the 244 sources selected for full-text screening, 181 were excluded including 10 where full-text could not be obtained by institutional subscriptions and/or contacting the author. The full-text articles were categorised according to type: research (170), grey literature (47), and opinion pieces (27). Each reviewer assessed articles from all categories to ensure a consistent approach.

The authors identified 14 systematic reviews but none addressed the same question as to the current scoping review. We screened these for relevant primary sources of which all but one were duplicates, and the remaining one was later removed as it did not meet the inclusion criteria.

Sixty-one remaining articles were included for data extraction using a data extraction tool to ensure a systematic approach. A further 10 sources were excluded. A final set of 53 sources was included in the scoping review, consisting of 35 research papers, 10 opinion papers, and 8 grey literature sources.

### Charting the data

Key items from each source were charted using a uniform approach and including author, setting/country, methodology, sample size, aims/objectives, and key findings (Table 1). Sources were categorised by the type of research; then papers from academic journals that did not include primary research such as theoretical and opinion papers; and lastly, grey literature. All sources were coded according to whether the source suggested that opioids were being underprescribed, overprescribed, demonstrated complex prescription patterns, or had no explicit stance.

### Collating, summarising and reporting the results

**Figure 3.**
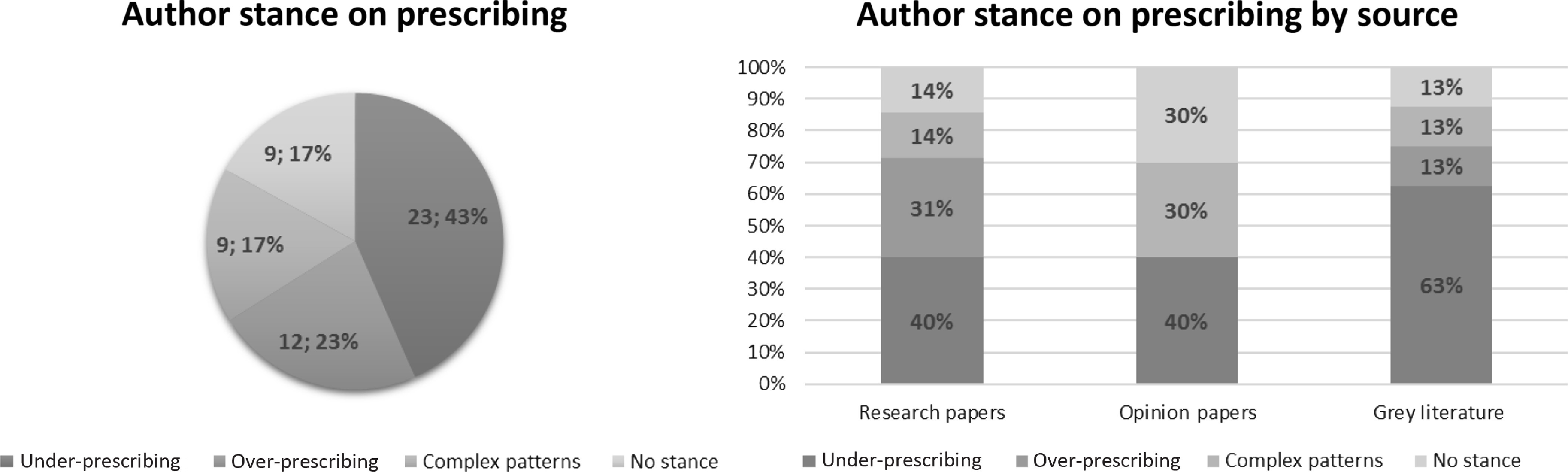
Author stance on prescribing overall and based on the source type

A substantial proportion of papers (*n* = 23, 43%) suggested that opioids were being under-prescribed for older adult pain management. However, the patterns diverged depending on the source. While the same proportion of research papers (40%) and opinion papers (40%) suggest under-prescribing is an issue, this figure was much higher in the grey literature (63%; grey literature was comprised predominantly of opinion pieces in practitioner-oriented non-academic periodicals). Overall, less than a quarter of papers identified over-prescribing as an issue, with no opinion papers addressing the over-prescription of opioids (see Figure 3).

Factors that were associated with prescribing patterns were categorised according to whether they were patient-related, prescriber-driven or system-driven. Table 2 demonstrates that patient factors including age, gender, race, and cognition appeared to influence prescribing decisions by physicians. However, prescriber factors were also important and included demographic characteristics such as the age of the prescriber themselves; attitudes towards the use of opioids, abuse/dependency, and on pain *per se*; and aspects of casework such as the number of contacts with the same patient. Policy/system factors were set in the context of the changing policy landscape over the last three decades. A key factor was funding criteria for medical care, particularly in the USA. However, system factors were rarely captured and seldom discussed.

**Table 2.** Factors influencing opioid prescribing for older adults by ‘factor source’

Patient Factors
Age; Gender; Race; Cognitive function/dementia; Learning Difficulties; Education; Type of pain; Alcohol/substance use; Functional status; Prior diagnosis of psychiatric illness; Prior use of psychiatric medication; Patient reluctance to take opioids; Joint pain; Location (community versus long-term care); Polypharmacy (use of multiple medications); Presence of arthritis; History of fractures; History of cancer; History of congestive heart failure; History of osteoporosis; Use of sedatives
Prescriber Factors
Age; Perception of pain as normal with ageing; Subjective judgements of ‘tolerable’ pain; Education on pain and prescribing; Failure to assess pain; Uniformity and speed of pain assessment; Attitudes on opioid prescribing; Attitudes on addiction; Fear of causing harm/concerns of opioid abuse or dependence; Not considering the initial use of weaker opioids; Confidence in pain management skills; Greater number of visits to the same patient; Physician type (e.g., attending, primary care); Familiarity with research confirming treatment benefits; Anticipated side-effects
Policy/System Factors
Administrative burden of prescribing; Availability of validated tools assessing opioid dosing for comorbidities; New laws/policy of prescribing; Funding eligibility for care; Institutional restrictions

The findings were also categorised as to whether the factors were associated with under-prescribing of opioids for older adult pain management, over-prescribing had no apparent effect or the findings were contradictory (see Table 3). There was considerable disagreement between some sources, for example, while three studies found that women were prescribed more opioids than men, one study found the reverse and another that gender had no effect. This demonstrates that opioid prescribing patterns are highly contextual depending on the setting, the period in time, and the interplay with other factors.

**Table 3.** Factors influencing opioid prescribing for older adults by prescribing trend

Factors Associated with Lower Opioids Prescribing
Older age (both compared to younger adults and among older adult age groups); Older age of the prescribing physicians; Intellectual disability; Perception of pain as normal for ageing; Self-funding of nursing care; Alcohol consumption; Failure to assess pain; Subjective judgements of ‘tolerable’ pain; Quick and simple verbal assessments of pain even for patients with cognitive impairment; Better functional status; ‘Conservative’ physician attitudes regarding opioids; Patient history of substance use; Administrative burden of prescribing; Prior diagnosis of psychiatric illness; Physician fear of causing harm/concerns of abuse or dependence; Patient/family reluctance to take opioids; Lack of suitable pain assessment tools; Anticipated side-effects
Factors Associated with Higher Opioid Prescribing
Lack of attempt to use weaker analgesics; Severe joint pain; Positive physician attitudes towards opioids; Greater physician confidence in pain management skills; Greater number of physician visits; Living in a nursing home; Polypharmacy; Prescriptions by an attending physician; Prescriptions by a primary care physician; Rheumatoid arthritis; Prior use of psychiatric medication; Patient/family education; Familiarity with research confirming treatment benefits; Validated tools assessing opioid dosing for comorbidities; Palliative care settings; Musculoskeletal pain and ‘pain not otherwise specified’; History of fractures; History of osteoporosis; Taking sedatives; History of cancer; History of congestive heart failure
Factors that Have No Influence on Opioid Prescribing
Level of pain; Charlson Comorbidity Index; Placement of the physicians (nursing home-based versus office-based)
Factors with Conflicting Findings
Female gender: three studies found an increase in prescribing, one a decrease, and one no effect Male gender: one study found increased prescribing for males, one study found decreased prescribing for males Race: two studies found lower prescribing for African Americans, one study found no influence of race or ethnicity Cognitive impairment/dementia/Alzheimer’s disease: one study found an increased prescribing of strong opioids only, five studies found decreased prescription of opioids New laws on prescribing: two studies found low prescribing due to deprescribing policy/scrutiny by regulatory agencies, one study found no effect of deprescribing policies over a longer period of time GP education: one study found lower prescribing due to increased GP education, two studies found a lack of GP knowledge associated to lower prescribing Functional status: one study found better functional status associated with lower prescribing, one study found impaired functional status associated with insufficient prescribing, one study found poor functional status associated with greater prescribing

## Discussion

**Figure 4.**
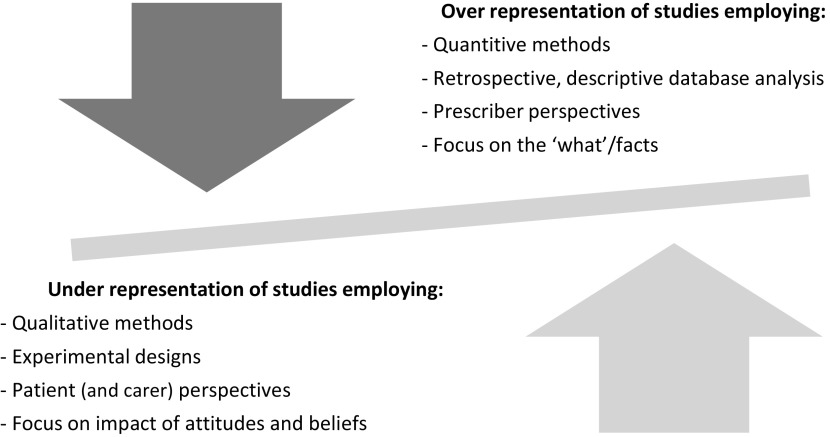
The characteristics of existing research on opioid prescribing for pain relief in older adults

The scoping review has identified a current imbalance in the literature exploring factors that influence opioid prescribing for older people for regular pain management (see Figure 4). Quantitative studies are more common, most often including secondary data analysis of prescribing databases. Research is also mainly descriptive rather than experimental, with a couple of notable exceptions (Shugarman *et al.*, 2010; Roxburgh *et al.*, 2011). More is known about *what* the influencing factors are, rather than *why* or *how* they operate. For example, while research shows that the patient’s age plays a role in opioid prescribing, it remains unclear why and how it affects prescriber decision-making. It is, for instance, possible that age is construed by the prescriber as an indication of comorbidities and age-specific risks of opioids (Siciliano, 2006), or it may stem from a belief that pain is a natural part of ageing (Niemi-Murola *et al.*, 2007). Research primarily considering attitudes and beliefs is lacking. Finally, while current research demonstrates that both patient and prescriber characteristics are influential in prescribing decisions, most research comes from the prescriber’s perspective and gives comparatively little attention to the perspectives of patients and carers, for example, their opinions on GP prescribing decisions.

While there are a number of existing systematic reviews in relation to opioid prescribing, these do not address all the intersections of the current review [i.e., looking at (1) influencing factors on (2) opioid prescribing for (3) pain management in (4) older people]. Some reviews have looked at potentially inappropriate prescribing for older people (Cherubini *et al.*, 2012; Cullinan *et al.*, 2014), but did not specifically address opioids, while others looked at pain management with any pain medicines (Kaye *et al.*, 2010), or focused on treating a specific subset of pain (e.g., acute pain; Fitzgerald *et al.*, 2017). In the one case of a review looking into opioid prescribing for older people (Huang and Mallet, 2013), it did not address factors influencing prescribing, but instead informed on best practice around opioid prescribing for older people.

### Strengths and limitations

To our knowledge, this is the first scoping review to date combining literature on factors influencing trends in opioid prescribing for older people. The scoping review methodology, which allowed for the inclusion of grey literature and non-research/commentary papers, has also significantly mitigated the issues around publication bias prominent in systematic reviews. This review also prioritised capturing the full scope of knowledge and illuminating knowledge gaps. It benefitted from multiple raters, which involved academics with experience in scoping and systematic reviews, as well as a practitioner (GP) with extensive knowledge of the topic, who reviewed ongoing findings.

Scoping reviews do not rate the quality or level of evidence provided therefore recommendations for practice cannot be graded; the aim is to provide a *broad* overview and identify gaps in the evidence. This approach avoided favouring academic perspectives over that of practitioners and allowed us to capture differing discourse trends within types of literature, for example, that under-prescribing was discussed more commonly in grey literature compared to academic sources.

A drawback of the review was that for a very small number of sources (*n* = −10) full-text articles could not be obtained (despite contacting the authors). An equally small (*n* = 9) number of non-English papers could not be assessed. As is true for most reviews, available sources did not include literature from the global south and disproportionately captured North American and European perspectives. In a similar way, the identification of relevant sources was predominately digital, with limited opportunities to hand-search sources, which may not be entered into online databases and cannot be found via online search engines.

### Implication for research and practice

The scoping review demonstrates that the policy climate significantly influences opioid prescribing for older adults (Siciliano, 2006; Cook, 2016). However, many of the studies were set in the US healthcare market and are unlikely to explain current GP opioid prescribing patterns for older adults in the UK. In addition to this, there was a notable lack of literature exploring the trajectories of opioid prescribing after initiation. Most studies explored the initial decision to prescribe but did not look at *when, how*, and *why* opioid prescribing becomes routine (i.e., a repeat prescription), dosages increase/decrease, and prescriptions are discontinued or changed for another type of opioid. There was also a gap in how prescribing decisions were perceived by older people and their carers (Closs *et al.*, 2004; Boerlage *et al.*, 2008; Green *et al.*, 2013) in particular their views on long-term use of opioids.

The UK-specific research needs to consider current prescribing policies and explore the prescriber and patient perspective. Additionally, the impact of knowledge, attitudes and beliefs around opioids, pain, and older people (held both by clinicians and by patients/carers) should be explored within the context of multi-morbidity and treatment burden. While there are few attitudinal studies to date and none are based in the UK, these studies suggest that ageism, in particular, may play a significant role (Kaasalainen *et al*, 2007; Niemi-Murola *et al.*, 2007). Research adopting a qualitative approach is needed to capture the complexity of interactions between patient, clinician, and system factors and this learning should be used to inform GP training and practice development.
